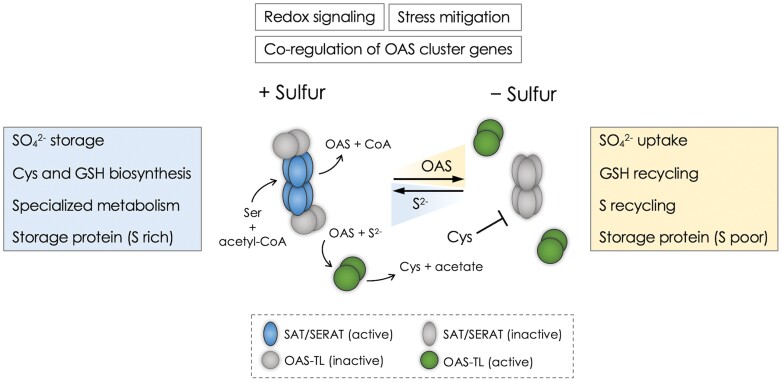# Sulfur metabolism: actions for plant resilience and environmental adaptation

**DOI:** 10.1093/jxb/erad164

**Published:** 2023-06-06

**Authors:** Hideki Takahashi, Frédéric Marsolais, Ann Cuypers, Stanislav Kopriva

**Affiliations:** Department of Biochemistry and Molecular Biology, Michigan State University, East Lansing, MI, USA; Genomics and Biotechnology, London Research and Development Centre, Agriculture and Agri-Food Canada, London, Ontario, Canada; Department of Biology, Western University, London, Ontario, Canada; Environmental Biology, Centre for Environmental Sciences, Hasselt University, Diepenbeek, Belgium; Institute for Plant Sciences, Cluster of Excellence on Plant Sciences, University of Cologne, Cologne, Germany

**Keywords:** Heavy metal, metabolism, redox, signaling, sulfur, transport

**Sulfur homeostasis is of vital importance for plant health and human nutrition. Sulfur nutrition strongly impacts plant resilience and nutritional quality traits in agricultural crop production. Recent discoveries provide dynamic perspectives on biological processes and mechanistic models delineating biochemical diversity and genetic regulation of plant sulfur metabolism. Invited reviews and articles in this Special Issue highlight current knowledge and ongoing research in sulfur biology.**

In the sulfur cycle in nature, plants are producers of the essential amino acids cysteine (Cys) and methionine (Met), both of which are incorporated into storage proteins serving as a dietary source consumed by animals (Takahashi *et al.*, 2011). Sulfur-containing metabolites and cofactors play pivotal roles in both abiotic and biotic stress mitigation processes, manifesting the importance of sulfur nutrition in plant resilience: for example, they contribute to redox signaling, heavy metal detoxification, and chemical defense against pathogen attack and herbivory in the environment. These topics were part of discussions at the 12th International Plant Sulfur Workshop held in London, Ontario, Canada in 2022, and are the basis for this Special Issue.

Plants monitor the environment and translate key information on external stimuli to intrinsic signals to alter gene expression, protein function, and protein stability, and take correct actions against the environmental stressors. We find intricate but tight connections between sulfur metabolism and cell signaling. Sulfide (S^2–^) and *O*-acetylserine (OAS), the substrates for Cys biosynthesis, modulate formation of the Cys synthase complex and sulfate assimilation (Hell and Wirtz, 2011; Sun *et al.*, 2021; Apodiakou and Hoefgen, 2023; Wirtz *et al.*, 2023) (Box 1). S^2–^ and OAS can also modulate the activity of the target of rapamycin (TOR) complex which optimizes plant growth (Dong *et al.*, 2017). Glutathione (γ-glutamylcysteinylglycine; GSH), the tripeptide synthesized from Cys, and Fe–S clusters impact the redox status (Caubrière *et al.*, 2023; Ito and Ohkama-Ohtsu, 2023; Iven *et al.*, 2023; Pedroletti *et al.*, 2023). Sulfation of metabolites and peptides generates 3’-phosphoadenosine-5’-phosphate (PAP), a retrograde signaling molecule communicating messages relevant to a wide spectrum of abiotic stress factors (e.g. excess light, drought, and oxidative stress) (Chan *et al.*, 2019). Biosynthesis and subcellular transport dynamics of these metabolite signals continue to represent intriguing questions about cell signaling over time and space.

## Thiols in action

Soil contaminants, such as arsenic (As), cadmium (Cd), lead (Pb), and mercury (Hg), are significant threats to global food security, deteriorating plant performance and human health. Plants exposed to these toxic metals and metalloids detoxify them through conjugation, sequestration, and avoidance mechanisms (Clemens and Ma, 2016). In this Special Issue, Sun *et al.* (2023) introduce As and Cd detoxification mechanisms, addressing what specific conjugants and transporters are involved and how they are mechanistically related to sulfur metabolism. Their recent work on the rice arsenite-tolerant mutant *astol1* and the causal dominant mutation which eliminates the catalytic activity of chloroplast-localizing *O*-acetylserine(thiol)lyase (OAS-TL) highlights the importance of the proposed sulfur-sensing function of the Cys synthase complex in As tolerance (Sun *et al.*, 2021). Their findings suggest the assembly of the Cys synthase complex—stabilized and activated to function as Ser acetyltransferase (SAT or SERAT) in the mutant—promotes sulfate uptake, reductive sulfur assimilation, and subsequent Cys and GSH biosynthesis, thus contributing to As detoxification (Sun *et al.*, 2021).

These toxic metals and metalloids are detrimental to plant health and thus may impact general cellular and organellar responses. Iven *et al.* (2023) extend discussion on how GSH and PC pools alter in the event of plant exposure to Cd and influence the ethylene and reactive oxygen species (ROS)-mediated signaling networks. Ramification of stresses imposed by Cd appears to involve ethylene and ROS signaling communicating with mitochondrial retrograde signaling, unfolded protein response, and autophagy. The authors further introduce potential actions of S^2–^ in plant Cd responses. As the product of sulfate assimilation and Cys catabolism, the potential signaling function of this highly reactive small molecule draws increasing attention. The prevalence of its biological impact may well be explained by translational and post-translational regulations associated with protein persulfidation (Aroca *et al.*, 2017). S^2–^ impacts the activity states and assembly/disassembly of the Cys synthase complex (Hell and Wirtz, 2011), altering the GSH pool size and signaling networks governing cellular responses to the redox status (Box 1).

Ito and Ohkama-Ohtsu (2023) highlight GSH metabolism and recycling pathways influencing the GSH pool size. GSH is synthesized from Cys in the chloroplast and partially in the cytosol by two-step reactions catalyzed by γ-glutamylcysteine synthetase and GSH synthetase. GSH forms conjugates with xenobiotics and endogenous bioactive metabolites including phytoallexins. γ-Glutamylpeptidases subsequently process these conjugates in the cytosol and vacuoles. Aside from these canonical aspects, the authors’ recent findings point to the concept of sulfur economy: γ-glutamylpeptidase and γ-glutamyl cyclotransferase are differentially expressed to enable GSH recycling, and GSH serves for conjugating glucosinolate breakdown products when there is a demand for sulfur recycling under sulfur deficiency (Sugiyama *et al.*, 2021; Ito *et al.*, 2022).

## Cys biosynthesis and metallocofactors

Cys biosynthesis is essential to protein synthesis in three subcellular compartments, the cytosol, chloroplasts, and mitochondria (Hell and Wirtz, 2011). SAT and OAS-TL exist in all three subcellular compartments. The rate of Cys biosynthesis depends on provision of two substrates—S^2–^ from the sulfate assimilation pathway in the chloroplast and OAS which derives from the amino acid Ser. The Cys synthase complex, the multienzyme complex of SAT and OAS-TL, functions not only as the catalyst for OAS biosynthesis but also for sulfur sensing. The suggested action mechanism for sulfur sensing involves assembly of the enzyme complex promoted by S^2–^ and its dissociation triggered by OAS (Hell and Wirtz, 2011) (Box 1).

Wirtz *et al.* (2023) present new data on this topic. By testing the impact of overexpression of enzymatically active and inactive forms of SAT in tobacco plants, they provide evidence that SAT overexpression enhances sulfate assimilation and Cys biosynthesis regardless of the SAT enzyme activity—the phenotype probably associated with stable Cys synthase complex formation encouraged in both the cytosol and chloroplasts. Interestingly, only the SAT overexpression in chloroplasts (with both the active and inactive forms of SAT) appears causative of chloroplast dysfunction and consequential downfall of the photosynthetic capacity. The impact of Cys overproduction on photosynthesis appears compartment specific and may require interacting partners specific to the Cys synthase complex in chloroplasts (Müller *et al.*, 2017).

Sulfur-containing cofactors play pivotal roles in metabolism (Caubrière *et al.*, 2023; Pedroletti *et al.*, 2023). Fe–S clusters are undoubtedly the most prominent and indispensable units, as they are essential for the photosynthetic and respiratory electron transfer chains (Balk and Schaedler, 2014). They are also essential for key enzyme functions in nitrogen and sulfur assimilation pathways, and synthesis of other important cofactors, such as lipoic acid, biotin, and thiamine. The molybdenum cofactor (Moco) is another essential metallocofactor required for nitrogen assimilation as well as ureide biosynthesis, and it is also involved in sulfite oxidation and abscisic acid (ABA) biosynthesis. Thus, the Fe–S cluster and Moco biogenesis is of crucial importance to the photosynthesis and basic metabolic function.

Cys desulfurase is the enzyme which is central to the extraction of sulfur from Cys, facilitating subsequent sulfur transfer or ‘sulfur trafficking’ in the Fe–S cluster and Moco biogenesis pathways. Caubrière *et al.* (2023) discuss functional diversity of Cys desulfurases in plants based on structural features and isoform-specific roles of this enzyme in three subcellular compartments, the cytosol, chloroplasts, and mitochondria. They further explore biological implication and possible evolutionary origins of Cys desulfurases coupling with Fe–S cluster assembly enzymes, sulfur trafficking enzymes, and thioredoxins involved in sulfide formation and redox regulations.

Fe–S cluster formation in mitochondria parallels its essentiality in chloroplasts. Pedroletti *et al.* (2023) provide detailed analyses of mitochondrial Fe–S cluster assembly mechanisms and machineries introducing sulfur to lipoic acid and biotin biosynthesis. They also mention possible impacts of loss of mitochondrial Fe–S clusters and target proteins and consequences of turnover of Fe–S cluster proteins. The article provides critical insights into the mitochondrial SAT function. Given the major contribution (~80%) of the mitochondrial SAT to the overall OAS production (Watanabe *et al.*, 2008; Hell and Wirtz, 2011), scavenging of S^2–^ released after disassembly of Fe–S clusters could rely on Cys biosynthesis locally in this organelle.

## Impact of OAS and sulfur nutrition

OAS is arguably the compound gaining traction over the past few decades in this research area. While it serves as a substrate for Cys biosynthesis, it is also recognized categorically as a signaling molecule triggering dissociation of the Cys synthase complex (Box 1). OAS accumulates under sulfur deficiency because of the appearance of SAT and OASTL, the enzymes responsible for Cys biosynthesis, at the juncture of sulfur assimilation and Ser biosynthetic pathways. Along these lines, OAS has been studied as a diagnostic molecular marker for plant sulfur status and an effector modulating sulfur deficiency response.

Apodiakou and Hoefgen (2023) provide an overview of the ‘OAS cluster genes’ co-expressed or co-regulated by sulfur deficiency and OAS in Arabidopsis. The robustness of gene regulatory networks of OAS cluster genes shown in this work corroborates the earlier findings of co-regulation of sulfur-responsive genes by the SULFUR LIMITATION 1/ETHYLENE INSENSITIVE 3-LIKE 3 (SLIM1/EIL3) transcription factor (Maruyama-Nakashita *et al.*, 2006; Aarabi *et al.*, 2016). The additional evidence shown based on comparative transcriptome analyses and DNA affinity purification (DAP) sequencing for SLIM1/EIL3 further emphasizes the role of this master regulator in sulfur nutritional response. Proposed interactive contributions of other transcription factors for sulfur and OAS responses await further investigations (Rakpenthai *et al.*, 2022).

Sulfur nutrition not only impacts Cys and Met biosynthesis, but also the ‘sulfur richness’ of seed storage proteins. Bonnot *et al.* (2023) discuss sulfur balancing mechanisms, the impact of abiotic stress factors on seed sulfur content and quality, and possible strategies for seed quality trait improvement. The authors address the importance of Met recycling through the *S*-methyl-Met cycle as a possible strategy to improve seed quality. They further mention the OAS cluster gene regulations in developing seeds of pea and wheat grains (Bonnot *et al.*, 2020; Henriet *et al.*, 2021), highlighting the impact of sulfur input and the role of the nuclear-localizing regulatory protein SULFUR DEFICIENCY INDUCED 1 (SDI1) in optimization of seed storage protein compositions, which extends the idea of sulfur homeostasis with this regulatory protein beyond its previously established function to inhibit the aliphatic glucosinolate biosynthesis in Arabidopsis (Aarabi *et al.*, 2016, 2021).

## Future directions

Sulfur metabolic enzymes are co-regulated to fulfill the demand for sulfur and stress mitigation. The concept of sulfur economy applies to balancing of primary and secondary metabolism, recycling of sulfur metabolites and cofactors, and alterations in seed storage protein compositions (Sugiyama *et al.*, 2021; Bonnot *et al.*, 2023; Ito and Ohkama-Ohtsu, 2023; Pedroletti *et al.*, 2023). The assembly/disassembly dynamics of the Cys synthase complexes with the actions of S^2–^ and OAS would impact sulfur metabolism and recycling pathways (Box 1). Beside the suggested direct impact of sulfur input and demands, movement of S^2–^ and OAS across subcellular compartments may also be decisive factors for this sensing mechanism. Co-regulation of the OAS cluster genes further signifies the OAS actions in sulfur signaling. The potential modularity of SLIM1/EIL3 in transcriptional regulation reveals important questions about interactions between sulfur demand signaling and mechanisms that control basic cell functions.

Studying plant–microbe and plant–insect interactions may offer new research directions. Plants are capable of mounting resistance mechanisms against pathogens and herbivores. Sulfur-containing specialized metabolites may be part of these plant defense mechanisms, as they are pre-emptively synthesized, stored, and metabolized into active forms to fend off pathogens and herbivores (Jacoby *et al.*, 2021). Pathogens, on the other hand, can deliver mechanisms to target sulfate transport and metabolism of host plant systems to compete for sulfur nutrients (Cernadas *et al.*, 2014; Wang *et al.*, 2022). Specialist insects co-evolved with host plants to evade plant defense mechanisms (Ratzka *et al.*, 2002; Okamura *et al.*, 2022). Our endeavors to explore these mechanistic frameworks continue to build new chapters in sulfur biology research in the face of grand challenges in crop production.

**Box 1. Simplified model illustrating dynamic assembly of Cys synthase complex and regulation of sulfur metabolism in plants**

Under sulfur-replete conditions (+Sulfur), S^2–^ promotes the assembly of the Cys synthase complex. Ser acetyltransferase (SAT/SERAT) hexamers and *O*-acetylserine(thiol)lyase (OAS-TL) dimers form this multienzyme complex. SAT/SERAT hexamers within the Cys synthase complex (blue) are free from feedback inhibition by Cys and remain active to synthesize OAS from Ser. OAS-TL dimers within the Cys synthase complex are catalytically inactive (gray). Free OAS-TL dimers (green) catalyze synthesis of Cys from OAS and S^2–^. Under sulfur-deficient conditions (–Sulfur), S^2–^ gradually becomes unavailable and OAS accumulates in the cell. OAS promotes dissociation of the Cys synthase complex. SAT/SERAT hexamers dissociated from the complex are inactive (gray) because they are sensitive to feedback inhibition by Cys. Illustration modified based on work by Hell and Wirtz (2011).

Boxes highlight responses to changes in sulfur conditions: under sulfur-replete conditions (left), plants accumulate SO_4_^2–^ and synthesize Cys, GSH, and sulfur-containing specialized metabolites. These metabolites contribute to maintaining the redox status and stress mitigation. The seed storage protein composition will become sulfur rich. Under sulfur-deficient conditions (right), plants activate SO_4_^2–^ uptake and promote sulfur recycling. Sulfur-rich seed storage proteins will become less abundant and replaced by sulfur-poor storage proteins. The OAS cluster genes are co-regulated and play important roles in these processes.